# Effects of bihemispheric transcranial direct current stimulation on motor recovery in subacute stroke patients: a double-blind, randomized sham-controlled trial

**DOI:** 10.1186/s12984-023-01153-4

**Published:** 2023-02-27

**Authors:** Shih-Pin Hsu, Chia-Feng Lu, Bing-Fong Lin, Chih-Wei Tang, I-Ju Kuo, Yun-An Tsai, Chao-Yu Guo, Po-Lei Lee, Kuo-Kai Shyu, David M. Niddam, I-Hui Lee

**Affiliations:** 1 Institute of Brain Science, National Yang Ming Chiao Tung University, Taipei City, Taiwan; 2 Division of Cerebrovascular Diseases, Neurological Institute, Taipei Veterans General Hospital, No. 201, Sec. 2, Shipai Rd., Beitou District, Taipei City, 11217 Taiwan; 3 Department of Biomedical Imaging and Radiological Sciences, National Yang Ming Chiao Tung University, Taipei City, Taiwan; 4 Department of Neurology, Far Eastern Memorial Hospital, New Taipei City, Taiwan; 5 Department of Neurosurgery, Taipei Veterans General Hospital, Taipei City, Taiwan; 6 Institute of Public Health, National Yang Ming Chiao Tung University, Taipei City, Taiwan; 7 Department of Electrical Engineering, National Central University, Taoyuan, Taiwan; 8 Brain Research Center, National Yang Ming Chiao Tung University, Taipei City, Taiwan

**Keywords:** Dual, Functional connectivity, Motor, Neuroplasticity, Stroke, tDCS, Upper extremity

## Abstract

**Background:**

Bihemispheric transcranial direct current stimulation (tDCS) of the primary motor cortex (M1) can simultaneously modulate bilateral corticospinal excitability and interhemispheric interaction. However, how tDCS affects subacute stroke recovery remains unclear. We investigated the effects of bihemispheric tDCS on motor recovery in subacute stroke patients.

**Methods:**

We enrolled subacute inpatients who had first-ever ischemic stroke at subcortical regions and moderate-to-severe baseline Fugl-Meyer Assessment of Upper Extremity (FMA-UE) score 2–56. Participants between 14 and 28 days after stroke were double-blind, randomly assigned (1:1) to receive real (n = 13) or sham (n = 14) bihemispheric tDCS (with ipsilesional M1 anode and contralesional M1 cathode, 20 min, 2 mA) during task practice twice daily for 20 sessions in two weeks. Residual integrity of the ipsilesional corticospinal tract was stratified between groups. The primary efficacy outcome was the change in FMA-UE score from baseline (responder as an increase ≥ 10). The secondary measures included changes in the Action Research Arm Test (ARAT), FMA-Lower Extremity (FMA-LE) and explorative resting-state MRI functional connectivity (FC) of target regions after intervention and three months post-stroke.

**Results:**

Twenty-seven participants completed the study without significant adverse effects. Nineteen patients (70%) had no recordable baseline motor-evoked potentials (MEP-negative) from the paretic forearm. Compared with the sham group, the real tDCS group showed enhanced improvement of FMA-UE after intervention (*p* < 0.01, effect size *η*^2^ = 0.211; responder rate: 77% vs. 36%, *p* = 0.031), which sustained three months post-stroke (*p* < 0.01), but not ARAT. Interestingly, in the MEP-negative subgroup analysis, the FMA-UE improvement remained but delayed. Additionally, the FMA-LE improvement after real tDCS was not significantly greater until three months post-stroke (*p* < 0.01). We found that the individual FMA-UE improvements after real tDCS were associated with bilateral intrahemispheric, rather than interhemispheric, FC strengths in the targeted cortices, while the improvements after sham tDCS were associated with predominantly ipsilesional FC changes after adjustment for age and sex (*p* < 0.01).

**Conclusions:**

Bihemispheric tDCS during task-oriented training may facilitate motor recovery in subacute stroke patients, even with compromised corticospinal tract integrity. Further studies are warranted for tDCS efficacy and network-specific neuromodulation.

*Trial registration*: This study is registered with ClinicalTrials.gov: (ID: NCT02731508).

**Supplementary Information:**

The online version contains supplementary material available at 10.1186/s12984-023-01153-4.

## Introduction

The randomized controlled trials (RCTs) of transcranial direct current stimulation (tDCS) applied to the primary motor cortex (M1) concurrently with rehabilitation therapy at the subacute stroke stage (> 7 days to 3 months) [1, 2], and even acute stage (1–7 days) [3–5], have demonstrated safety and potentials to promote motor recovery and neuromodulation of the underlying cortex [6]. However, clinical translation of tDCS has been limited by heterogeneity in stroke lesions and tDCS setups across studies, so their effects on the augmentation of stroke recovery remain undetermined. Anodal low intensity (0.5–2 mA) stimulation usually increases corticospinal excitability [7], while cathodal stimulation may decrease corticospinal excitability but have substantial inter-subject variability and partially non-linear dose-dependent effect in healthy subjects [7–10]. Although the mechanisms of motor recovery after stroke are largely unclear, there are indications that mild-to-moderate patients with the good outcome have residual ipsilesional M1 corticospinal excitability [11–13]. In contrast, patients with severe hemiplegia have purely over-excitability of the contralsional M1 [13–15]. In the recent meta-analysis, three stimulation types have been compared with multi-session tDCS after stroke using Fugl-Meyer Assessment of Upper Extremity (FMA-UE) as the primary efficacy outcome [16]: including (1) anodal tDCS over the ipsilesional M1 (with the cathode placed over the contralesional supraorbital region; eight RCTs with one acute, three subacute and four chronic stages), (2) cathodal tDCS over the contralesional M1 (with the anode placed over the ipsilesional supraorbital region; four RCTs with two subacute, one chronic and one mixed stage), and (3) bihemispheric (or dual) tDCS with the anode over the ipsilesional M1 and the cathode over the contralesional M1; seven RCTs with six chronic and one mixed stage. Among the stimulation types, bihemispheric tDCS seemed to have a relatively large effect size on motor recovery of the paretic upper extremity (UE) [16], mostly in chronic patients more than six months post-stroke [17–21]. However, the efficacy of bihemispheric tDCS for UE recovery in subacute or acute stroke patients remains unclear [3, 5, 22, 23]. Few studies reported insignificant effects for UE recovery in acute stroke patients [5, 22], while the other recent study found beneficial effects in combination with modified constraint-induced movement therapy in acute-to-subacute stroke patients with mild motor impairment [23]. Specifically, there is good evidence that the subacute stroke phase coincides with a window of spontaneously enhanced neuroplasticity from preclinical and clinical studies [24, 25]. Applying bihemispheric tDCS during this critical window of enhanced neuroplasticity may be especially important to investigate.

Many factors may influence the tDCS effects on post-stroke motor recovery [16, 26], including the corticospinal tract (CST) integrity, adjunct therapy, and stimulation timing and doses. The CST integrity, measured by means of transcranial magnetic stimulation with the motor-evoked potential (MEP) recorded from the paretic UE, is a prognostic biomarker of post-stroke motor outcome and a predictive biomarker for tDCS responsiveness [27, 28]. Patients with preserved MEPs were usually responders to intensive training compared with those patients without MEPs [29, 30]. However, most tDCS RCTs have not stratified this key element at baseline [3, 5, 17–21]. Furthermore, adjunct therapy coupled with task-oriented training [17, 18, 31], rather than with simple joint exercise [19, 20], helped to improve UE motor functions. As for stimulation timing, concurrent tDCS with training resulted in better effects than tDCS applied before conventional therapy [16]. Finally, dose-dependent stimulation with a higher current density or charge density has been suggested to enhance greater post-stroke motor improvement in the meta-analyses of multi-session tDCS RCTs [16, 32]. However, possible non-linear effects of tDCS in stroke patients shall also be considered. Collectively, we hypothesized that multi-session bihemispheric tDCS during task-oriented training would provide therapeutic potential in subacute stroke patients, even those with compromised CST integrity.

Here, we stratified stroke patients by the paretic UE severity and CST integrity, utilizing a double-blind, randomized, sham-controlled design to elucidate the effects of multi-session task-concurrent bihemispheric tDCS on domain-specific FMA-UE and FMA-Lower Extremity (FMA-LE), Action Research Arm Test (ARAT). Moreover, we explored neural correlates underlying bihemispheric tDCS on the targeted sensorimotor network using resting-state functional magnetic resonance imaging (fMRI) in these subacute stroke patients. Our previous study demonstrated that a single-session bihemispheric tDCS simultaneously modulated bilateral corticospinal excitability in subacute stroke patients with preserved MEPs, in which electrophysiological changes were predicted by the baseline contralesional-to-ipsilesional transcallosal inhibition ratio between the M1s [33]. Post-stroke resting-state functional connectivity (FC) between the M1s was shown to be positively correlated with better motor recovery [34–37]. Hence, we hypothesized that bihemispheric tDCS might modulate interhemispheric and/or intrahemispheric FC of the target M1s, respectively, in correlation with tDCS-induced FMA-UE change scores. Addressing the lack of studies on FC changes by bihemispheric tDCS after stroke, our results can provide insights into the neuromodulation of functional networks by non-invasive brain stimulation.

## Materials and methods

### Study design and participants

**Fig. 1:**
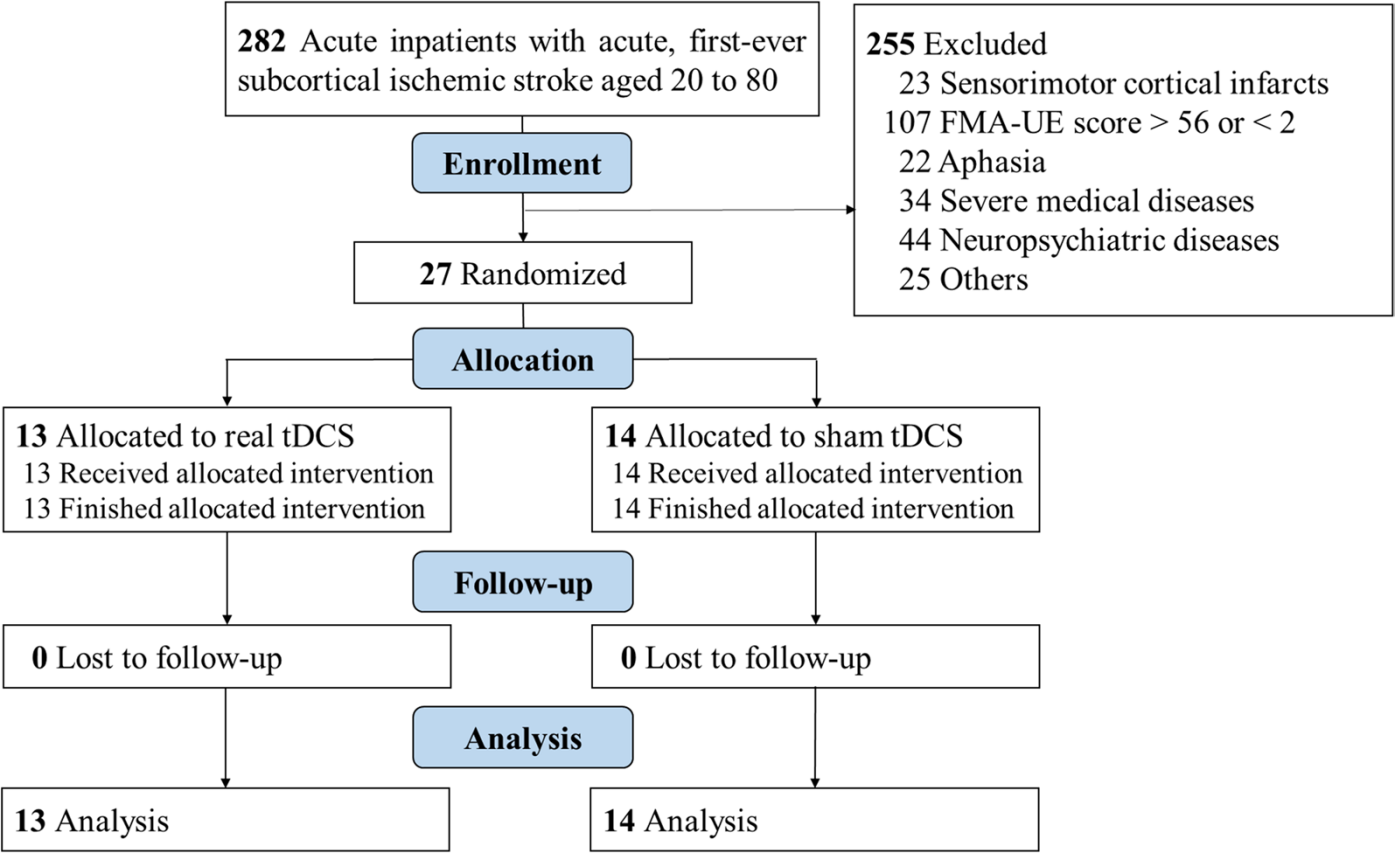
Enrollment flowchart of this randomized controlled trial. FMA-UE, Fugl-Meyer Assessment of Upper Extremity; tDCS, transcranial direct current stimulation

We conducted a double-blind, randomized, sham-controlled study to investigate the efficacy and safety of bihemispheric tDCS for motor recovery in subacute stroke patients. The ethics committee approved the study at the Taipei Veterans General Hospital (VGHIRB No. 2015-03-003C) and registered with ClinicalTrials.gov (NCT02731508). We screened 282 consecutive inpatients between September 2015 and June 2021 and validated their eligibility for the following inclusion criteria (Fig. 1): (1) age between 20 and 80 years; (2) acute first-ever unilateral infarction confirmed by diffusion-weighted MRI; (3) consciousness clear and able to sign the informed consent form. The exclusion criteria were: (1) sensorimotor cortical infarcts; (2) too mild or too severe FMA-UE scores, i.e. > 56 or < 2 (0–66, where 0 is no function and 66 is maximum) [38]; (3) sensory or motor aphasia; (4) severe medical diseases (advanced malignancy, end-stage heart, liver or kidney failure, etc.) with premorbid modified Rankin Scale (mRS) > 1; (5) major neuropsychiatric diseases (dementia, epilepsy, parkinsonism, cerebellar ataxia, major depression, etc.); (6) contraindications to transcranial magnetic stimulation (TMS) for increased risk (presence of metallic implants, pregnancy); and (7) participating in other interventional studies.

### Sample size estimation

The G*Power software (v3.1.9.4; Franz Faul, University of Kiel, Kiel, Germany) was used for sample size estimation. Based on a previous study using 15 sessions of bihemispheric tDCS during occupational therapy in stroke patients (current density: 0.08 mA/cm^2^; total stimulation time: 450 min) [21], the effect size on the improvement of FMA-UE was 1.4. If the effect size was assumed as Cohen’s *d* = 1.4 (equal to Pearson’s *r* = 0.57 for nonparametric statistics), the estimated sample size was n = 11 per group to achieve a statistical power of 80% and a 2-tailed test with α = 0.05. Given a possible drop-out rate of 15%, at least 13 participants for each group were required.

### Randomization, concealment, stratification and blinding

We randomly assigned the eligible participants within 2–4 weeks after stroke onset to ensure the groups were matched for baseline UE impairment. Computerized randomization minimization was used to balance the stratification factors between groups [39], including age, lesioned side, baseline FMA-UE score [40], and the ipsilesional CST integrity [27]. To determine the residual CST integrity, single-pulse TMS was administrated using a Rapid^2^ stimulator (Magstim, Whitland, UK) with a double-cone coil (126 mm in diameter) after a safety screening as previously described [33]. An absence of MEP recorded from the paretic extensor carpi radialis (MEP-negative) was defined as a lack of potentials with an amplitude of at least 50 μV when using the maximal stimulator output. The randomization process was conducted by a person who was unaware of the study hypotheses. Both the participants and the assessors who performed outcome measures were blinded to group allocation.

### Bihemispheric tDCS intervention

**Fig. 2:**
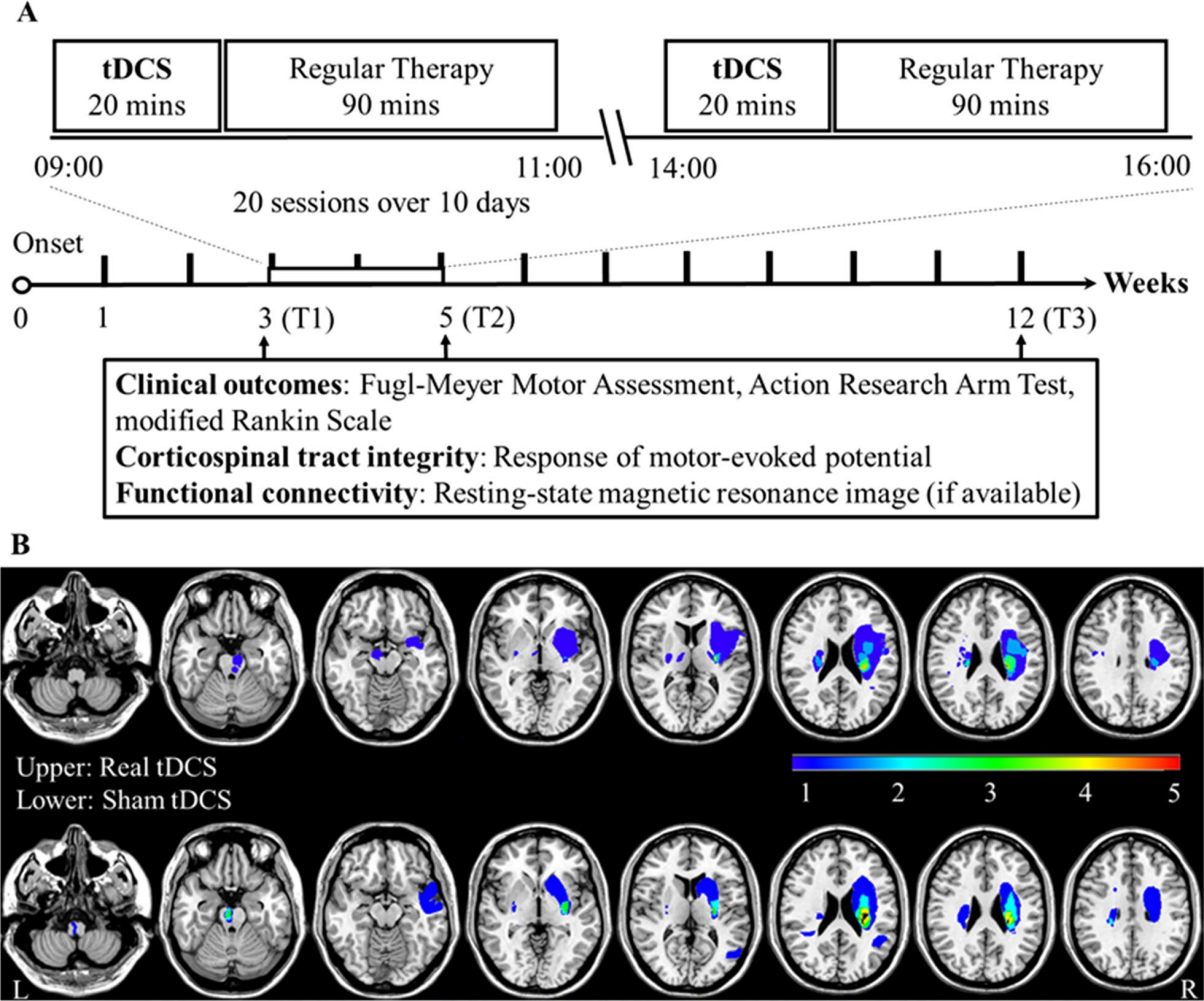
Schematic overview of the study protocol and lesion overlap map. **A** All participants received 20 sessions of 20-min real or sham bihemispheric transcranial direct current stimulation (tDCS) with concurrent task-oriented therapy before 90-min conventional regular rehabilitation twice-daily for ten weekdays. The interventional timeline included longitudinal assessments at three timepoints (T1, T2, and T3). **B** Diffusion-weighted MRI-identified infarct maps of each group (real 13 vs. sham 14) overlaid on a standard brain from the Montreal Neurological Institute. The color bar indicated the participant number

A NeuroConn DC stimulator (Ilmenau, Germany) was used to deliver direct current through two conductive rubber electrodes wrapped in normal saline-soaked sponges (5 × 5 cm^2^) with wires placed towards the posterior head. The real tDCS group received 20 min (including ramp-up and ramp-down for 30 s each) of 2-mA tDCS (current density 0.08 mA/cm^2^) with anodal and cathodal electrodes placed over the ipsilesional and contralesional M1 of the hand, respectively, based on anatomical C3 and C4 locations (e.g. the 10–20 system) as previously described [33]. Sessions were conducted prior to regular conventional therapy twice daily for ten workdays (total stimulation time: 400 min; Fig. 2A). The sham tDCS settings were similar except that the direct current ceased after 2 min (including 30 s ramp-up) to keep blindness that the session started with real direct current to induce participants habituated to the tDCS-induced feelings on the scalp. During 20-min tDCS (or sham), the participant simultaneously practiced occupational therapist-led UE tasks, including shoulder and scapular movements, elbow flexion and extension, forearm supination and pronation, wrist movements, or grasp and release objects, tailored to meet individualized mobility and goals following the principles of task-oriented therapy [41] with minimal physical support if possible. For paralyzed muscle groups, training was initiated using single-joint tasks with eliminated gravity position, followed by anti-gravity position and multi-joint tasks when possible. The researchers (SPH and IJK) were responsible for the administration of tDCS intervention at the bedside and interviewing participants for any adverse events [42]. Afterwards, all inpatients received 90-min sessions of regular conventional therapy twice daily, including occupational therapy (UE range of motion exercise and strengthening, hand skill training, and balance training) and physical therapy (lower extremity mobility and strengthening, aerobic exercise, and gait training) before discharge.

### Primary and secondary outcomes

Based on the tDCS target of UE M1 with electrode locations over C3 and C4, the primary efficacy outcome was the change in FMA-UE scores assessing UE mobility [38], including a proximal subscale (0–42) for the shoulder/elbow/forearm and a distal subscale (0–24) for the wrist/hand. The minimal clinically important difference (MCID) for change in FMA-UE has been suggested as 10 points after intervention [43], which we used to define treatment responders [29]. The secondary outcomes included changes in the Action Research Arm Test (ARAT) assessing UE activity (0–57, maximum score 57) [44], Fugl-Meyer Assessment of Lower Extremity (FMA-LE) for lower extremity mobility (0–34, maximum score 34) [38], mRS for global function after stroke (0–6) [45], and resting-state FC of the target network after intervention (see below). The FMA-LE was assessed for the occurrence of off-target effects. All above measurements were administered at three timepoints: pre-intervention baseline (T1), immediately post-intervention (T2), and three months post-stroke (T3) (Fig. 2A).

For the safety outcome, the Adverse Effects Questionnaire was used after each tDCS session, which includes 11 questions about occurrence of headache, neck pain, scalp pain, tingling, itching, burning sensation, skin redness, sleepiness, trouble concentrating, acute mood change, or other specified conditions [42].

### Brain MRI and resting-state fMRI acquisition and preprocessing

Brain images were acquired with a 3.0-T GE Discovery 750 MRI scanner (GE Healthcare, Chicago, IL). Participants were asked to keep their eyes open without thinking or moving during the scan. A standard head coil (eight channels) with foam padding was used to restrict head motion. All imaging was acquired along the anterior–posterior commissural plane, as identified by multiplanar T1-weighted BRAVO anatomical images (repetition time, 12.2 ms; echo time, 5.2 ms; flip angle, 12°; voxel size, 1 × 1 × 1 mm^3^; field of view 256 × 256 mm^2^). For resting-state fMRI, blood oxygenation level-dependent (BOLD) signals from a task-free run were recorded with a T2* gradient-echo echo-planar imaging sequence (repetition time/echo time, 3000/30 ms; flip angle, 90°; field of view, 222 × 222 mm^2^; matrix size, 64 × 64; slice thickness, 3 mm; 47 slices; 124 volumes) as previously described [46]. The aforementioned scanning time was approximately 15 min. Participants were scanned at the aforementioned three timepoints if available (Fig. 2A).

Briefly, the fMRI data were preprocessed with Statistical Parametric Mapping (SPM12; https://www.fil.ion.ucl.ac.uk/spm/) in the following order: correction of slice timing (the 47^th^ slice as reference), realignment to the mean image for correcting head motion with a 6-parameter rigid-body transformation, flipping realigned BOLD images and anatomical T1 images for those with left hemispheric strokes to the right, coregistration of the mean BOLD image to the anatomical image, spatial normalization to the Asian brain template with affine registration, and smoothing using a 6-mm full-width half-maximum Gaussian kernel. The first four volumes of BOLD images were discarded from the subsequent analyses. Nuisance signals, including the six head movement parameters, the mean signal of cerebrospinal fluid, white matter, and global signal, were regressed out from the smoothed images, and low-frequency signals (0.01–0.1 Hz) were extracted using MATLAB software (2018b; Mathworks, Natick, MA) and an in-house scripts [47].

### Target regions of interest and seed-based analysis of functional connectivity

A region of interest (ROI)-to-ROI approach was adopted to investigate the resting-state sensorimotor network primarily. Twelve ROIs (with corresponding MNI coordinates) with 6-mm radii were predefined for the paretic hand representation from a meta-analysis of movement-related fMRI in 472 patients with various impairment from acute to chronic phase after ischemic stroke [13], including contralesional M1 (cM1, − 38, − 24, 58), ipsilesional M1 (iM1, 42, − 14, 52), contralesional S1 (cS1; − 36, − 30, 60), ipsilesional S1 (iS1; 40, − 28, 52), contralesional supplementary motor area (cSMA; − 4, − 6, 54), ipsilesional SMA (iSMA; 4, − 6, 54), contralesional dorsolateral premotor cortex (cPMd; − 42, − 10, 58), ipsilesional PMd (iPMd; 42, − 6, 56), contralesional ventrolateral premotor cortex (cPMv; − 46, − 10, 48), ipsilesional PMv (iPMv; 42, − 6, 48), contralesional anterior intraparietal sulcus (cIPS; − 42, − 40, 50), and ipsilesional IPS (iIPS; 42, − 40, 50). The 12 cortical ROIs were not overlapped with any subcortical lesions. Hence, we didn’t remove lesion voxels from individual ROIs. The averaged BOLD signals of all voxels in each ROI were extracted and ROI pairwise associations were calculated using Pearson’s correlation coefficients (*r*; 66 pairs in total among 12 ROIs). The FC strength between each ROI pair was then calculated as the transformed *r*-values (i.e. z-scores) using Fisher *r*-to-z transformation. The FC between ROIs was expressed as “FC_ROI-ROI_” and FC changes as “△FC_ROI-ROI_”. ROI pairs and their anatomical locations were visualized by means of BrainNet Viewer 1.7 (https://www.nitrc.org/projects/bnv/).

### Statistical analysis

An intention-to-treat procedure was used to deal with possible missing data. Analyses were performed using SPSS 24 (IBM, Armonk, NY) and MATLAB 2018b. The demographic and baseline characteristics were compared using a 2-sample *t*-test, Mann–Whitney *U* test, or *χ*^*2*^ test. After the normality test, we adopted the mixed-design, repeated measures analysis of covariance (ANCOVA) to exam the time, group, and the group-by-time interaction effects on primary and secondary outcomes, using the baseline score as a covariate in the ANCOVA [48] with a post hoc Bonferroni correction for multiple comparisons. The effect size of experimental tDCS was estimated using eta square (η^2^), where the large, medium, and small effect sizes η^2^ were set at 0.138, 0.056, and 0.01 [49], respectively. In addition, patients who had no recordable baseline MEP from the paretic wrist extensors were defined as MEP-negative participants who have compromised CST integrity and relatively poor prognosis [27]. Therefore, we conducted an MEP-negative subgroup analysis to test tDCS effects on their primary outcome using the aforementioned ANCOVA.

We performed a stepwise multivariate regression analysis to investigate the relationship between the altered functional sensorimotor network and the primary outcome of FMA-UE improvement (T2–T1 and T3–T1 as the dependent variables) in the real tDCS group and the sham group, respectively, as previously described [47]. To avoid an overfitting model, only the altered FC (z scores with large Cohen’s f^2^ > 0.5) estimated by simple linear regression for FMA-UE improvements were included in the multivariate regression model with adjustment for age and sex [49]. The performance of generated linear regression models was assessed by the goodness-of-fit (R^2^) and F statistic with *p* < 0.05 as significance. An independent variable was considered significant if *p* < 0.05. The amount of multicollinearity in a set of multiple regression variables was examined to remove redundant FC changes with variance inflation factor > 10 [50]. Finally, we compared the tDCS-related connectivity changes between groups (T2–T1 and T3–T1) using the 2-sample *t*-test with Bonferroni correction. For intra-group changes over time, the significant regressors (FC changes) for the FMA-UE improvement from the multivariate regression model were examined using the paired t-test with Bonferroni correction.

## Results

### Participant characteristics

Twenty-seven eligible participants (mean age [standard deviation]: 59.2 [11.4] years, 15 males, all right-handed, 15 with right hemispheric infarcts, mean baseline FMA-UE 31.2 [18.8]) were randomly assigned to receive real tDCS (n = 13) or sham stimulation (n = 14). All participants completed the 3-month clinical follow-up (Fig. 1). The baseline demographic and neurophysiological characteristics were comparable between groups (Table 1). Nineteen participants (70.4%; real n = 9 vs. sham n = 10) had no recordable MEP from the paretic wrist extensors across all timepoints, except for one person in the real group and one person in the sham group who regained the MEP at post-intervention and three months post-stroke, respectively. MRI showed that participants of both groups primarily had subcortical infarctions along the CST, particularly at the corona radiate and the internal capsule (n = 21), or the ventral brainstem (n = 6) (Fig. 2B).

**Table 1:** Baseline characteristics of early subacute stroke participants

	Real tDCS, n = 13	Sham tDCS, n = 14	*p*
Age (years)			
Mean (SD)	59.1 (11.4)	59.2 (11.8)	0.98
Sex, n (%)			
Male	9 (69.2)	6 (42.9)	0.17
Lesion side, n (%)			
Right	9 (69.2)	7 (50)	0.31
Stroke onset to tDCS (days)			
Mean (SD)	20.7 (3.5)	21.1 (5.3)	0.83
Functions, mean (SD)			
NIHSS	5.9 (3.1)	6.4 (3.7)	0.75
mRS	3.5 (1.1)	3.3 (1.3)	0.79
FMA-UE	31.8 (17.5)	30.4 (20.8)	0.86
ARAT	18.9 (15.3)	17 (18.2)	0.78
FMA-LE	20.5 (7.4)	22.3 (8.3)	0.57
Corticospinal tract status			
MEP-, n (%)	9 (69.2)	10 (71.4)	1.0
Lesion volume (mL)			
Mean (SD)	5.4 (10.2)	5.1 (10.1)	0.82
Lesion location, n (%)			
Subcortical	11 (84.6)	10 (71.4)	0.72
Brainstem	2 (15.4)	4 (28.6)	
Thrombolysis received			
n (%)	3 (23.1)	2 (14.3)	0.93

*ARAT* Arm Research Action Test, *FMA-UE/LE* Fugl-Meyer Motor Assessment of Upper Extremity/Lower Extremity, *MEP-* absence of motor evoked potentials recorded from the paretic extensor carpi radialis, *mRS* modified Rankin Scale, *NIHSS* National Institute of Health Stroke Scale, *SD* standard deviation, *tDCS* transcranial direct current stimulation

The doses of regular rehabilitation after intervention were comparable between groups. Seven and eight participants in the real (54%) and sham (57%) groups, respectively, continued high-frequency hospitalized rehabilitation (4–5 days per week) until three months post-stroke, while the other six participants in both the real (46%) and sham (43%) groups maintained a low-to-moderate frequency of outpatient rehabilitation (≤ 3 days per week) until three months post-stroke.

### Safety and blindness

All participants tolerated 20 sessions of real or sham tDCS without significant adverse events, comparable to the recent review [51]. There were three participants in the real group and two in the sham group that reported tingling feelings, and two participants in each group reported itching. One participant receiving real tDCS showed transient redness of the scalp at the anodal site. The risks of the aforementioned events were similar between groups (*p* = 1.0). The success of blinding status was assessed post-intervention: two and three participants in the real and sham group, respectively, assumed themselves to be receiving a placebo.

### Bihemispheric tDCS during task-oriented therapy promoted motor recovery after stroke

**Fig. 3:**
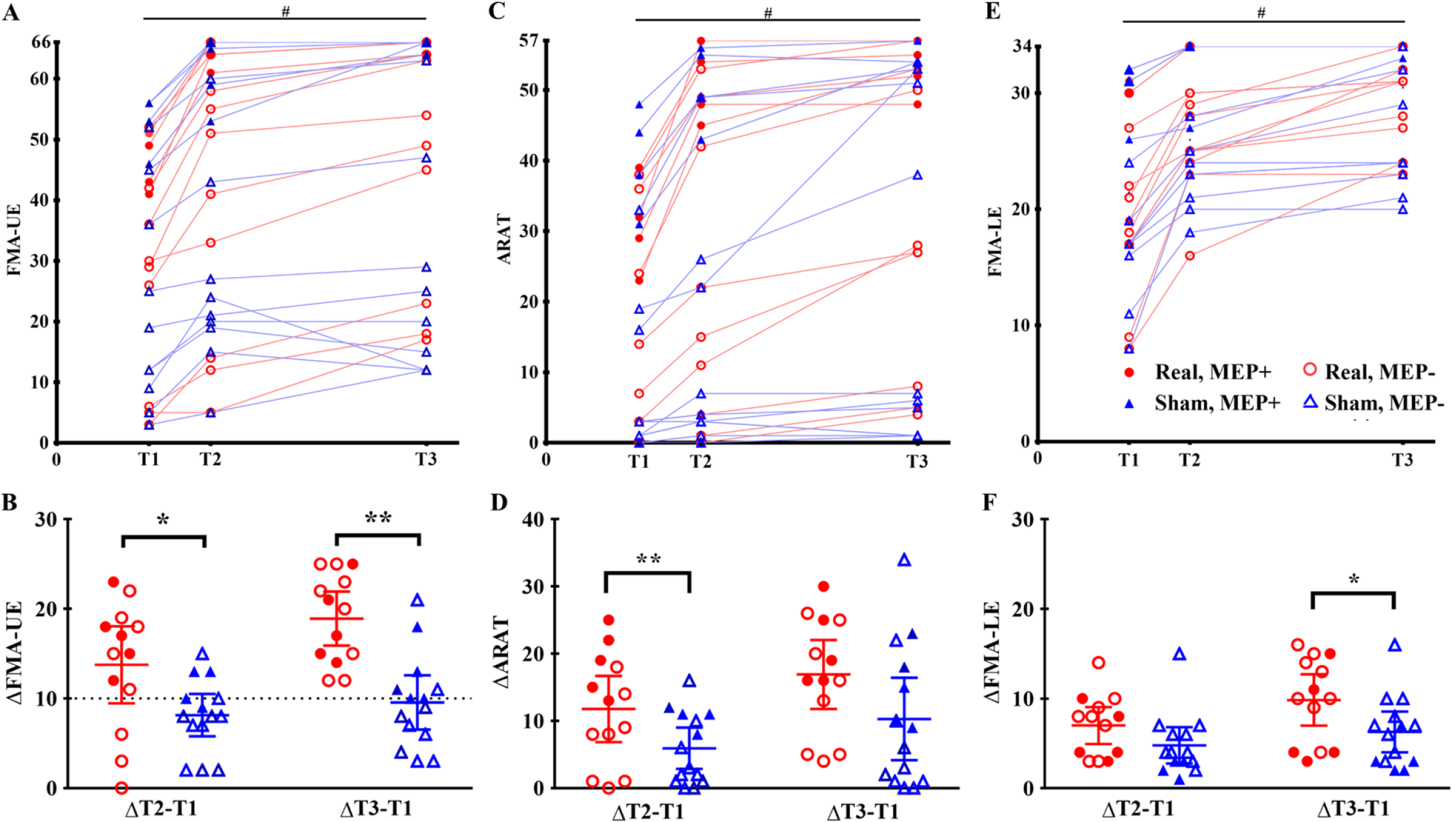
Motor recovery after bihemispheric transcranial direct current stimulation (tDCS) during task-oriented training in subacute stroke patients. **A** The individual trajectory of the Fugl-Meyer Assessment of Upper Extremity (FMA-UE) scores with significant time effects within both groups in the early subacute phase. Red circles and blue triangles represent real and sham tDCS groups, respectively. Solid and hollow symbols indicate participants with and without forearm motor evoked potentials (MEP ±), respectively. **B** The real tDCS group showed significantly better FMA-UE improvements than the sham group after the 2-week intervention and at three months post-stroke. The dashed line shows the minimal clinically important difference of FMA-UE = 10 points to define treatment responders. **C** The individual trajectory of the Action Research Arm Test (ARAT) scores with significant time effects within both groups. **D** The ARAT improvements of real group were greater than those the sham group after the intervention. However, this significant difference did not last to the 12 weeks post-stroke. **E** The individual trajectory of the Fugl-Meyer Assessment of Lower Extremity (FMA-LE) scores with significant time effects within both groups. **F** The FMA-LE improvements after intervention were not different between groups until 12 weeks post-stroke. ^#^ *p* < 0.001, compared with the baseline using repeated measures ANCOVA (Table 2); * *p* < 0.025 (Bonferroni correction: 0.05/2 timepoints), ** *p* < 0.005, compared with the sham group

The primary efficacy outcome of FMA-UE improvements showed significant time effect (*p* < 0.001), group effect (*p* < 0.001) and group-by-time interaction effect (*p* < 0.001, η^2^ = 0.327) (Table 2). After a post hoc analysis, the real tDCS group demonstrated greater increases in FMA-UE scores after the 2-week intervention (mean difference [95% confidence interval]: real 13.5 [9.1–17.8] vs. sham 8.4 [5.8–10.5]; *p* = 0.018, η^2^ = 0.211) and at three months post-stroke (real 19.1 [15.9–22.2] vs. sham 9.4 [6.3–12.5]; *p* < 0.001, η^2^ = 0.522), respectively, than the sham group (Fig. 3A, B). Notably, in the MEP-negative subgroup analysis (real n = 9 vs. sham n = 10), their FMA-UE improvements also showed significant time effect (*p* < 0.001), group effect (*p* = 0.004), and group-by-time effects (*p* = 0.002, η^2^ = 0.332). The real tDCS MEP-negative subgroup had a greater long-term improvement at three months post-stroke compared with the sham MEP-negative subgroup (real 18.9 [14.9–22.8] vs. sham 8.1 [4.4–11.8]; *p* < 0.001, η^2^ = 0.545), but not immediately after the 2-week intervention (real 11.3 [7.1–15.5] vs. sham 6.9 [2.9–10.9]; *p* = 0.156, η^2^ = 0.122), with a reduced sample size and power. Overall, the real group had a higher probability of being responders after intervention (real 76.9% vs. sham 35.7%; *p* = 0.031) and sustained the effect until 3 months post-stroke (real 100% vs. sham 50%; *p* = 0.012) (Fig. 3B).

**Table 2:** The primary and secondary motor outcome measures after the real versus sham tDCS

	Real tDCS			Sham tDCS			rmANCOVA, *p*-value (η^2^)		
	T1	T2	T3	T1	T2	T3	Time	Group	Group-by-Time
*Primary outcome*									
FMA-UE	31.8 (17.5)	45.4 (22.3)	50.9 (19.3)	30.6 (20.5)	38.8 (22.1)	40.2 (24.1)	< 0.001 (0.392)	< 0.001 (0.452)	** < 0.001** **(0.327)**
*Secondary outcomes*									
ARAT	18.9 (15.3)	30.8 (22.1)	36.1 (20.5)	17.0 (18.2)	22.9 (22.7)	27.3 (25.5)	0.002 (0.285)	0.012 (0.234)	0.062 (0.122)
FMA-LE	20.5 (7.5)	27.5 (5.2)	30.3 (3.8)	22.3 (8.3)	27.1 (5.9)	28.6 (5.6)	< 0.001 (0.81)	0.007 (0.264)	**0.013** **(0.165)**

*ARAT* Arm Research Action Test, *FMA-UE/LE* Fugl-Meyer Assessment of Upper extremity/Lower Extremity, *rmANCOVA* mixed-design, repeated measures analysis of covariance, *T1* baseline, *T2* post-intervention, *T3* 12 weeks after onset. The values represent the mean (standard deviation)

The secondary outcome of ARAT improvements showed significant time effect (*p* = 0.002) and group effect (*p* = 0.012), but not group-by-time interaction effect (*p* = 0.062) with a medium effect size (η^2^ = 0.122, Table 2). Both groups had significant ARAT improvements in parallel after the 2-week intervention (mean difference [95% confidence interval]: real 11.9 [7.1–16.7] vs sham 5.9 [2.9–9]; group difference: *p* = 0.004, η^2^ = 0.294) and at 3 months post-stroke (real 17.2 [12.2–22.2] vs sham 10.3 [4.2–16.4]; group difference: *p* = 0.06, η^2^ = 0.139). However, the inter-group difference at three months post-stroke became insignificant, which needs further and larger studies (Fig. 3C, D). Likewise, the secondary outcome of FMA-LE improvements exhibited significant time effect (*p* < 0.001), group effect (*p* = 0.007) and group-by-time interaction effect (*p* = 0.013, η^2^ = 0.165, Table 2). Compared with the sham group, the real tDCS group didn’t have greater increases in FMA-LE scores after the intervention (real 7.2 [5–9.3] vs. sham 4.8 [2.8–6.8]; *p* = 0.06, η^2^ = 0.139) until at 3 months post-stroke (real 9.9 [7–12.7] vs. sham 6.3 [4–8.6]; *p* = 0.006, η^2^ = 0.276, Fig. 3E, F), suggesting an indirect off-target effect. Although 100% of the participants after real tDCS had a favorable outcome (mRS 0–2) at three months in comparison to 76.9% of the sham group, there was no significant intergroup difference in the proportion (*p* = 0.25).

### Individual FMA-UE improvements after tDCS were associated with bilateral intrahemispheric, rather than interhemispheric, connectivity changes

Of the 27 participants, 23 (real 12 vs. sham 11) received baseline fMRI, 21 (real 11 vs. sham 10) completed the post-intervention scanning, and 19 (real nine vs. sham ten) finished the follow-up scanning. Dropouts for fMRI were due to claustrophobia, the COVID-19 pandemic, or technical problems. The baseline characteristics of the 23 patients who underwent fMRI did not differ from those of the 27 patients (*p* = 0.15–1.0), and there was no baseline difference between groups in the 23 patients. Among them, the significant time, group, and group-by-time effects remained (all *p* ≦ 0.001). Real tDCS group had greater FMA-UE improvements after the 2-week intervention (13.2 [9.4–16.7] vs. sham 7.6 [3.8–11.5]; *p* = 0.018) and at three months post-stroke (18.9 [15.7–22.2] vs. sham 8.5 [5.1–11.8]; *p* < 0.001), respectively. The FC analyses in the reduced subpopulation should therefore be representative of all 27 patients in this study.

**Fig. 4:**
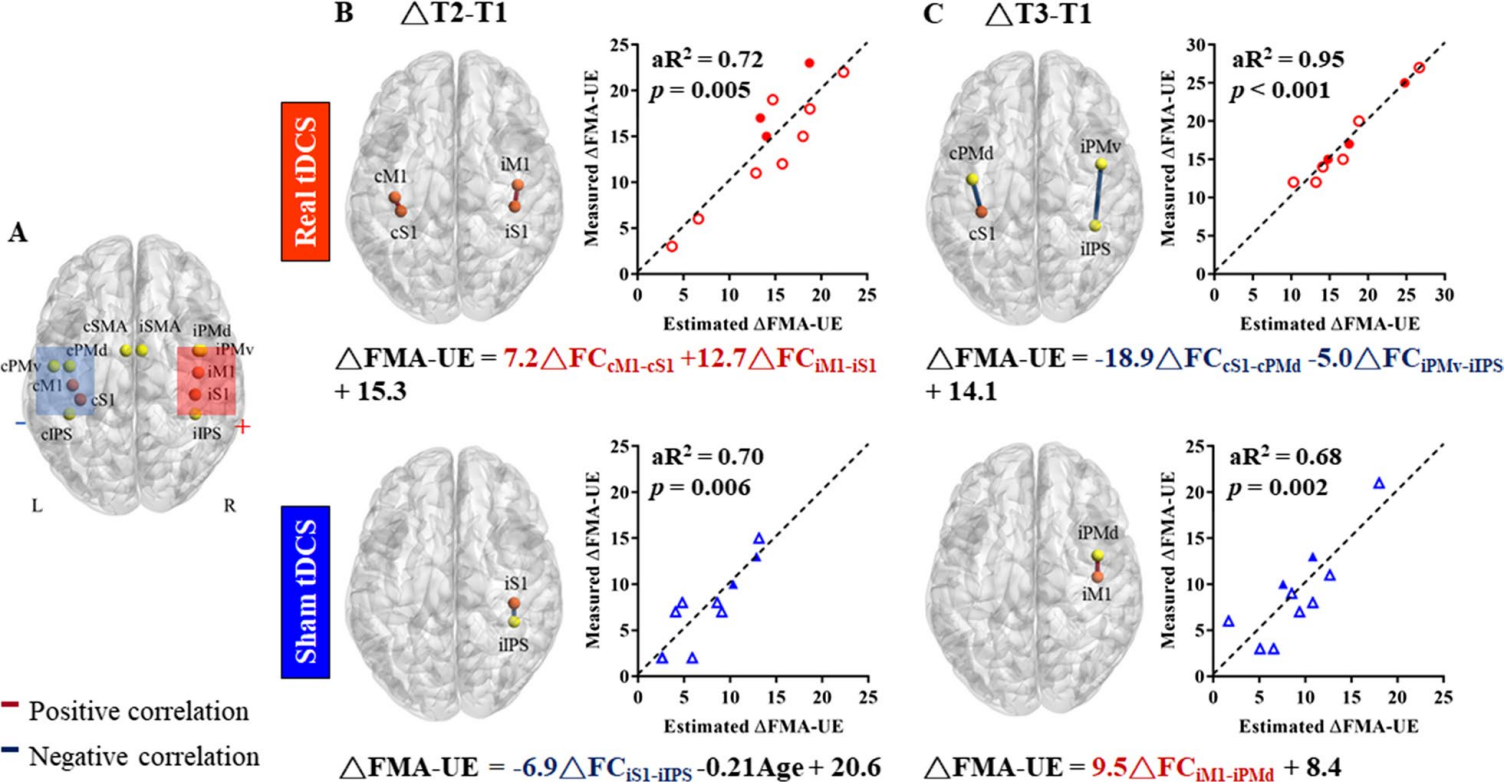
Target regions of interests (ROI) of the sensorimotor network and tDCS-related functional connectivity changes involved in motor recovery after stroke. **A** Anatomical illustration of the 12 ROIs. The orange circles are the primary sensorimotor cortices, while the yellow circles are explorative network hubs. Rectangles illustrate the projected placements of the anode (red) and cathode (blue). **B** The significant relationships between changes in functional connectivity (△FC) and changes in FMA-UE (△FMA-UE) after 2-week real versus sham tDCS (△T2–T1). Scatter plots represent the actual observed △FMA-UE (y-axis) and the estimated △FMA-UE (x-axis) from the multiple regression model. **C** The significant relationships between △FC and △FMA-UE after 12 weeks post-stroke (△T3–T1). Dark red lines connecting ROIs and △FC in formulas indicate positive correlations between △FC and △FMA-UE, whereas dark blue lines (△FC) indicate negative correlations. Circles and triangles represent the real and sham tDCS group, respectively. Solid symbols indicate participants with preserved motor evoked potential (MEP +), while hollow symbols indicate absent MEP (MEP −). aR^2^, adjusted R squared; tDCS, transcranial direct current stimulation; FMA-UE, Fugl-Meyer Assessment of Upper Extremity

On the individual-level of the tDCS-induced after effects, we linearly correlated 66 pairs of FC changes (△FC) with the concurrent FMA-UE changes, including 12 ROIs in the sensorimotor network (Fig. 4A) using stepwise multivariate regression analyses (Table 3, exclusion details of insignificant FC are listed in the Additional file 1: Table S1). Interestingly, after the 2-week real tDCS, individual △FC_cM1-cS1_ and △FC_iM1-iS1_ were positively and synergistically correlated with their FMA-UE improvement, which jointly explained 72% of the variance of UE motor recovery (adjusted R^2^ = 0.72, *p* = 0.005, Fig. 4B). By contrast, after the 2-week sham tDCS, only the ipsilesional △FC_iS1-iIPS_ was negatively correlated with their FMA-UE improvement (adjusted R^2^ = 0.45, *p* = 0.02), and additionally including age as an independent factor increased the prediction accuracy to 70% (adjusted R^2^ = 0.70, *p* = 0.006, Fig. 4B). The results suggest that spontaneous recovery following task training alone was related to age and ipsilesional connectivity changes, while enhanced recovery after concurrent bihemispheric tDCS likely involved intrahemispheric FC in bilateral hemispheres rather than interhemispheric FC changes. At three months post-stroke, individual △FC_cS1-cPMd_ and △FC_iPMv-iIPS_ after real tDCS were correlated with long-term FMA-UE improvement (adjusted R^2^ = 0.95, *p* = 0.00005, Fig. 4C), while only individual △FC_iM1-iPMd_ after sham tDCS was correlated with FMA-UE recovery (adjusted R^2^ = 0.68, *p* = 0.002). Age and sex did not significantly influence UE recovery at this phase.

**Table 3:** Multivariate regression model coefficients for FMA-UE improvement

Variable	B	SE	β	*p*	VIF
*From T1 to T2*					
Model for real group: R^2^ = 0.77 (aR^2^ = 0.72), p = 0.005					
△FC_cM1-cS1_	7.18	2.12	0.59	0.0094	1.08
△FC_iM1-iS1_	12.75	4.41	0.51	0.02	1.08
Constant	15.29	1.05		< 0.001	
Model for sham group: R^2^ = 0.77 (aR^2^ = 0.7), p = 0.006					
△FC_iS1-iIPS_	− 6.88	1.83	− 0.69	0.007	1
Age	− 0.21	0.08	− 0.51	0.028	1
Constant	20.57	4.84		0.004	
*From T1 to T3*					
Model for real group: R^2^ = 0.96 (aR^2^ = 0.95), p < 0.001					
△FC_cS1-cPMd_	− 18.89	2.19	− 0.82	< 0.001	1.39
△FC_iPMd-iIPS_	− 5.02	1.81	− 0.26	0.032	1.39
Constant	14.05	0.52		< 0.001	
Model for sham group: R^2^ = 0.72 (aR^2^ = 0.68), p = 0.002					
△FC_iM1-iPMd_	9.52	2.13	0.85	0.002	
Constant	8.39	0.96		< 0.001	

*aR*^*2*^ adjusted R squared, *cM1/iM1* contralesional/ispilesional primary motor cortex, *cPMd/iPMd* contralesional/ispilesional dorsal premotor cortex, *cS1/iS1* contralesional/ispilesional primary somatosensory cortex, *B* unstandardized regression coefficient, *β* standardized beta coefficient, *FMA-UE* Fugl-Meyer Assessment of Upper Extremity, *iIPS* ispilesional intraparietal sulcus, *SE* standard error, *VIF* variance inflation factor

At the group level, there were no intergroup differences in FC_iM1-iS1_, FC_cM1-cS1_, FC_iM1-cM1_, FC_iS1-iIPS_, FC_cS1-cPMd_, FC_iPMv-iIPS_, or FC_iM1-iPMd_ at baseline (Additional file 1: Table S2) after correction (all uncorrected *p* = 0.038–0.984, corrected threshold = 0.007). Also, there were no significant intragroup changes in △FC_iM1-iS1_, △FC_cM1-cS1,_ △FC_iM1-cM1_, △FC_iS1-iIPS_, △FC_cS1-cPMd_, △FC_iPMv-iIPS_, or △FC_iM1-iPMd_ over time after correction (all uncorrected *p* = 0.014–0.47, corrected threshold = 0.003), nor were there intergroup differences in the above FC changes after correction (all uncorrected *p* = 0.023–0.971, corrected threshold = 0.003, Additional file 1: Table S2), which were likely attributed to the small sample size and large inter-individual variations.

## Discussion

We demonstrated that, for the first time, concurrent bihemispheric tDCS during task-oriented training conferred benefits on motor recovery of the paretic UE, compared to sham stimulation in early subacute stroke patients. These individual UE improvements were associated with bilateral intrahemispheric FC changes in the targeted motor network. However, there was high variability of individual FC changes and no significant difference between groups. The results suggest that the neural circuits involved in tDCS-related subacute recovery are likely reorganized in the bilateral cortices. Importantly, our RCT of bihemispheric tDCS stratified patients according to the residual CST integrity, a prognostic biomarker for motor recovery [27]. Among the patients with compromised CST integrity (MEP-negative), the real tDCS group showed greater but delayed UE improvement than the sham group at three months post-stroke. The subcortical or brainstem infarctions along the CST may be compensated by circuits elsewhere in the sensorimotor network. Although the compensatory role of the contralesional M1 for or against interhemispheric competition remains elusive, depending on stroke severity and the ipsilesional CST integrity, early bihemispheric tDCS may promote immediate and lasting motor recovery after stroke.

Our findings of bihemispheric tDCS for UE motor recovery are in line with previous studies in chronic stroke patients [17, 21, 31]. Lindenberg et al. [17]. and Alisar et al. [21]. found that, using 5–15 sessions of similar bihemispheric tDCS settings during conventional therapy in chronic patients after three months post-stroke, the FMA-UE improvement rate was 20.7–35.2% (vs. 3.2–6.6% with sham stimulation) from baseline. In the present study with subacute patients around one-month post-stroke, the FMA-UE improvement rate after real tDCS was 42.5% (vs. 27.5% with sham stimulation) and it increased to 62.4% (vs. 29.6% with sham stimulation) at three months post-stroke. Furthermore, immediately after real tDCS, the mean FMA-UE improvement reached MCID (13.5), in contrast to those after sham tDCS (8.4). Taken together, our study implies that applying bihemispheric tDCS may safely augment the effects of subacute stroke rehabilitation, particularly clinically meaningful UE improvement.

The effect of bihemispheric tDCS on ARAT was not as prominent as FMA-UE, although there was greater improvement of ARAT immediately after real tDCS compared with sham tDCS. One explanation could be that ARAT has stronger floor effect and ceiling effect than FMA-UE in acute and subacute stroke patients [52, 53]. In other words, it renders ARAT unable to discriminate participants at either extreme of the scale. The ARAT items require more integral UE function of reaching and grasping compared to FMA-UE [54]. Specifically, a larger sample of 30 participants is recommended according to our estimated effect size (η^2^ = 0.122).

The mechanisms of bihemispheric tDCS for motor recovery in subacute stroke patients remain unclear. In our analysis of functional networks underlying motor recovery, bihemispheric tDCS-induced individual UE improvements were associated with intrahemispheric FC_iM1-iS1_ and FC_cM1-cS1_ changes bilaterally, while spontaneous UE improvements after sham stimulation were associated with ipsilesional FC_iS1-iIPS_ changes. These findings indicate that motor network reorganization at the near-stimulated regions possibly play a role of bihemipheric tDCS effects. In healthy subjects, bihemispheric tDCS over the M1s during a motor task has been shown to enhance motor learning accompanied by similarly increased BOLD signals [55] and regional cerebral blood flow [56] at the bilateral peri-rolandic regions. However, we did not observe significant inter-group differences of the FC changes following bihemispheric tDCS. Previous stroke studies have suggested that the increased resting-state interhemispheric FC_iM1-cM1_ positively correlated with spontaneous motor recovery after stroke [35–37]. Our patient selection of moderate-to-severe UE paresis with mostly compromised CST integrity [57] in early subacute stage [58] could partly explain this discrepancy.

There are limitations of this study. First, because of the enrollment criteria for relatively homogenous stroke patients, the small sample size weakened external validity and further resting-state FC changes and subgroup analysis of tDCS responsiveness by patient-specific factors [26, 33, 59]. It should be cautious to interpret our findings. However, we found that our protocol might have a delayed long-term benefit on UE motor recovery for patients with compromised CST integrity (MEP-negative). The MEP status assessment was performed using a biphasic waveform pulse with a Magstim Rapid^2^ system. An approach to verify the ipsilesional CST integrity might be to explore CST lesion load or fractional anisotropy to provide further confidence in the results [59, 60]. Second, the target ROI-based analysis might underestimate FC changes outside the ROIs, and the inter-individual difference in stroke lesions may mask the modulatory effect of tDCS on resting-state FC [61]. Further studies in large patient samples for the tDCS mechanisms are warranted. Finally, the 10–20 system anatomical landmarks for tDCS electrode positions (C3 and C4) may not be optimal for MEP-negative patients. Patient-tailored targets for tDCS modulation in those with compromised CST integrity needs to be further characterized. Movement-related electroencephalogram or functional near-infrared spectroscopy could be considered to guide electrode positions close to the hotspot [62].

## Conclusions

In summary, bihemispheric tDCS is a promising approach in combination with task-oriented training for facilitating motor recovery in subacute stroke patients, including those with compromised residual CST integrity. The neural underpinnings of simultaneous neuromodulation of bilateral M1s might be mediated by intrahemispheric connectivity reorganization of the bilateral sensorimotor network. Further studies are required to validate the current findings.

## Supplementary Information

**Additional file 1: Table S1. **The complementary information to Table 3 of insignificant functional connectivities after real or sham transcranial direct current stimulation (tDCS) in relation to individual FMA-UE improvements. They were excluded from the stepwise multivariate regression model by the level of significance. **Table S2. **Comparisons of resting-state functional connectivity (FC) changes at the transcranial direct current stimulation (tDCS)-related sensori-motor cortex after real versus sham intervention.

## Data Availability

The dataset used during this study are available from the corresponding author on reasonable request.

## Abbreviations

tDCS: Transcranial direct current stimulation
M1: Primary motor cortex
FMA-UE: Fugl-Meyer Assessment of Upper Extremity
ARAT: Action Research Arm Test
FMA-LE: Fugl-Meyer Assessment of Lower Extremity
FC: Functional connectivity
MEP: Motor-evoked potentials
RCT: Randomized controlled trial
UE: Upper extremity
CST: Corticospinal tract
fMRI: Functional magnetic resonance imaging
mRS: Modified Rankin Scale
TMS: Transcranial magnetic stimulation
MCID: Minimal clinical important difference
T1: Pre-intervention baseline
T2: Post-intervention
T3: Three months post-stroke
BOLD: Blood oxygenation level-dependent
ROI: Region of interest
cM1/iM1: Contralesional/ipsilesional primary motor cortex
cS1/iS1: Contralesional/ipsilesional primary somatosensory cortex
cSMA/iSMA: Contralesional/ipsilesional supplementary motor area
cPMd/iPMd: Contralesional/ipsilesional dorsolateral premotor cortex
cPMv/iPMv: Contralesional/ipsilesional ventrolateral premotor cortex
cIPS/iIPS: Contralesional/ipsilesional intraparietal sulcus
ANCOVA: Analysis of covariance
