# Supplementary material for: Effects of bihemispheric transcranial direct current stimulation on motor recovery in subacute stroke patients: a double-blind, randomized sham-controlled trial
Source: J Neuroeng Rehabil. 2023 Feb 27;20:27. doi: 10.1186/s12984-023-01153-4 (PMC9969953; doi:10.1186/s12984-023-01153-4)
Supplement: Supplementary file 1 — Additional file 1: Table S1. The complementary information to Table 3 of insignificant functional connectivities after real or sham transcranial direct current stimulation (tDCS) in relation to individual FMA-UE improvements. They were excluded from the stepwise multivariate regression model by the level of significance. Table S2. Comparisons of resting-state functional connectivity (FC) changes at the transcranial direct current stimulation (tDCS)-related sensori-motor cortex after real versus sham intervention. [file 12984_2023_1153_MOESM1_ESM.docx]

**Additional file**

**Table S1.** The complementary information to Table 3 of insignificant functional connectivities after real or sham transcranial direct current stimulation (tDCS) in relation to individual FMA-UE improvements. They were excluded from the stepwise multivariate regression model by the level of significance.

| **Real tDCS** | | | | **Sham tDCS** | | | |
| --- | --- | --- | --- | --- | --- | --- | --- |
| **ROI pairs** | **B** | **SE** | ***p*** | **ROI pairs** | **B** | **SE** | ***p*** |
| ***After intervention (△T2-T1)*** | | | |  | | | |
| cM1-cIPS | 0.89 | 4.01 | 0.83 | iS1-iSMA | -0.74 | 2.96 | 0.81 |
| cS1-cIPS | 0.37 | 4 | 0.93 | iS1-iPMd | 0.51 | 3.77 | 0.9 |
| iS1-cIPS | 1.79 | 5.79 | 0.77 | iS1-iPMv | -0.42 | 3.2 | 0.9 |
| iS1-iIPS | 0.61 | 3.73 | 0.88 | iSMA-iIPS | -0.21 | 0.08 | 0.8 |
| cPMd-cIPS | -0.3 | 5.58 | 0.96 |  |  |  |  |
|  |  |  |  |  |  |  |  |
| ***Three months post-stroke (△T3-T1)*** | | | | |  |  |  |
| cM1-cPMd | 1.91 | 1.67 | 0.32 | cM1-cPMv | 3.96 | 3.09 | 0.24 |
| cM1-iPMd | 0.89 | 2.2 | 0.51 | cM1-cIPS | 6.64 | 3.56 | 0.1 |
| iM1-iPMd | -2.99 | 1.52 | 0.12 | iM1-iS1 | 2.58 | 2.31 | 0.3 |
| cSMA-cPMd | -1.64 | 1.84 | 0.42 | iM1-iPMv | -7.18 | 4.9 | 0.19 |
| iSMA-iIPS | 0.97 | 0.69 | 0.24 | iM1-iIPS | 3.68 | 2.65 | 0.21 |
| cPMd-cPMv | 0.18 | 1.6 | 0.92 | cS1-cIPS | -3.84 | 3.92 | 0.36 |
| iPMd-iIPS | 0.86 | 2.3 | 0.01* | iS1-cSMA | -2.85 | 8.34 | 0.74 |
|  |  |  |  | iS1-iPMd | 2.47 | 3.32 | 0.48 |
|  |  |  |  | iS1-iPMv | -2.16 | 3.92 | 0.6 |
|  |  |  |  | iS1-iIPS | 4.49 | 2.33 | 0.1 |
|  |  |  |  | cSMA-iPMd | -1.52 | 3.06 | 0.63 |
|  |  |  |  | cSMA-iPMv | -4.04 | 3.41 | 0.28 |
|  |  |  |  | iSMA-cPMv | -5.27 | 4.66 | 0.3 |
|  |  |  |  | iPMd-iIPS | -1.95 | 5.11 | 0.71 |
|  |  |  |  | cPMv-cIPS | 2.64 | 2.68 | 0.36 |

ROI, region of interest; B, unstandardized regression coefficient; T1, baseline; T2, post-intervention; T3, three months after stroke onset; cIPS/iIPS, contralesional/ipsilesional intraparietal sulcus; cM1/iM1, contralesional/ipsilesional primary motor cortex; cPMd/iPMd, contralesional/ipsilesional dorsal premotor cortex; cPMv/iPMv, contralesional/ipsilesional ventral premotor cortex; iS1/cS1, contralesional/ipsilesional primary sensory cortex; iSMA/cSMA, contralesional/ipsilesional supplementary motor area; SE, standard error. * iPMv-iIPS removed from the model due to high multicollinearity (variance inflation factor = 12.1).

**Table S2.** Comparisons of resting-state functional connectivity (FC) changes at the transcranial direct current stimulation (tDCS)-related sensori-motor cortex after real versus sham intervention.

| **FC of ROI Pairs** | **Real tDCS** | **Sham tDCS** | **Intergroup**  ***p_uncorrected_*** |
| --- | --- | --- | --- |
| **iM1-cM1**, T1 | 0.39 (0.29) | 0.3 (0.3) | 0.448 |
| △T2-T1 | 0.07 (0.15) | 0.17 (0.36) | 0.547 |
| △T3-T1 | -0.11 (0.28) | 0.01 (0.42) | 0.415 |
| **iM1-iS1**, T1 | 0.8 (0.34) | 0.56 (0.33) | 0.105 |
| △T2-T1 | 0.02 (0.36) | 0.08 (0.49) | 0.728 |
| △T3-T1 | -0.25 (0.28)^#^ | 0.18 (0.13) | 0.061 |
| **cM1-cS1**. T1 | 1.23 (0.36) | 1.23 (0.21) | 0.984 |
| △T2-T1 | -0.15 (0.14) | -0.1 (0.44) | 0.807 |
| △T3-T1 | 0.03 (0.49) | -0.14 (0.17) | 0.466 |
| **iS1-iIPS,** T1 | 0.58 (0.42) | 0.54 (0.29) | 0.788 |
| △T2-T1 | -0.07 (0.36) | -0.01 (0.45) | 0.738 |
| △T3-T1 | -0.1 (0.52) | -0.09 (0.42) | 0.971 |
| **cS1-cPMd**, T1 | 0.65 (0.3) | 0.39 (0.27) | 0.038* |
| △T2-T1 | -0.1 (0.29) | 0.04 (0.21) | 0.224 |
| △T3-T1 | -0.16 (0.24)^#^ | 0.14 (0.3) | 0.023* |
| **iPMv-iIPS**, T1 | 0.37 (0.35) | 0.21 (0.26) | 0.223 |
| △T2-T1 | 0.03 (0.33) | -0.09 (0.35) | 0.42 |
| △T3-T1 | -0.06 (0.29) | 0.01 (0.34) | 0.66 |
| **iM1-iPMd**, T1 | 1.04 (0.49) | 0.9 (0.28) | 0.394 |
| △T2-T1 | -0.01 (0.28) | -0.16 (0.4) | 0.327 |
| △T3-T1 | -0.13 (0.25) | 0.07 (0.47) | 0.248 |

The mean of Z scores (standard deviation) represents FC strength.

ROI, region of interest; cM1/iM1, contralesional/ipsilesional primary motor cortex; cPMd/iPMd, contralesional/ipsilesional dorsolateral premotor cortex; cS1/iS1, contralesional/ipsilesional primary sensory cortex; iIPS, ipsilesional intraparietal sulcus; iPMv, ipsilesional ventrolateral premotor cortex; T1, baseline; T2, post-intervention; T3, three months after stroke onset. Between-group difference* by the 2-sample *t*-test and intra-group**^#^** by the paired *t*-test were all insignificant after Bonferroni correction (threshold = 0.003).
